# Attitudes towards and knowledge about Human Papillomavirus (HPV) and the HPV vaccination in parents of teenage boys in the UK

**DOI:** 10.1371/journal.pone.0195801

**Published:** 2018-04-11

**Authors:** Susan Mary Sherman, Emma Nailer

**Affiliations:** School of Psychology, Keele University, Keele, Staffs, United Kingdom; University of Toronto, CANADA

## Abstract

The incidence of cancers attributable to Human Papillomavirus (HPV) that affect males is on the rise. Currently in the UK teenage boys are not vaccinated against HPV while teenage girls are. The rationale for not vaccinating boys is that vaccinating girls should provide herd immunity to boys, however this does not protect men who have sex with men or men who have sex with unvaccinated women. The issue of whether to vaccinate boys or not is a controversial one with considerable lobbying taking place to change the existing policy. On one side of the debate are financial considerations while on the other side health equality is important. One avenue that has not been presented is the parental perspective. The current study uses a self-report questionnaire to explore what parents of teenage boys know about HPV and the vaccine and whether they want the vaccine for their sons. Only half of the parents had heard of HPV prior to completing the survey. Of those who had heard of HPV, knowledge about the health sequelae of HPV for men was poor relative to their knowledge about its impact on female health. Parents who would be willing to vaccinate their sons had higher levels of knowledge about HPV than those parents who would be unwilling or unsure. Irrespective of whether they had previously heard of HPV or not, once provided with a brief description of HPV, the majority of parents thought that boys should be offered the vaccination. There is a pressing need for public education about the potential impact of HPV on male health in order to facilitate uptake of the vaccine in the event of the vaccination programme being extended to men or to facilitate informed decision making about seeking the vaccine privately in the event that it isn’t.

## Introduction

By March 31^st^ 2017, 71 (37%) countries across the world had introduced the HPV vaccination as part of their vaccination schedule for girls. Just 11 of those countries had also introduced the vaccination for boys [1]. Currently in the UK boys are not vaccinated against HPV, while girls are, despite the rise of HPV-related cancers in men. While the main justification for this is financial, there are other important considerations.

Human papillomavirus (HPV), a sexually transmitted infection, is responsible for 99.7% of cases of cervical cancer [2] and 530,000 new cases of cervical cancer globally every year [1]. Most studies exploring knowledge and awareness of HPV have focused on knowledge about the link between HPV and cervical cancer and HPV and genital warts for which it is also responsible. However, in addition to causing cervical cancer and genital warts, HPV is also responsible for some head and neck cancers, some penile cancers and the majority of anal cancers [3] and the incidence of these cancers is on the rise. For example, the incidence of head and neck cancers attributable to the high-risk HPV types 16 and 18 in Europe, which is five-fold higher in men (12,707 new cases yearly) than women (2,531 new cases yearly), is increasing [4]. In the UK alone, the incidence of squamous cell carcinoma of the oral cavity and oropharynx in men rose by 51% between 1989 and 2006 [4,5]. Globally, HPV types 16 and 18 are responsible for 38,000 (85%) new cases of head and neck cancers and 35,000 (87%) cases of anal cancers [1].

Currently in the UK, girls aged 12–13 are routinely offered a free vaccination against HPV by the National Health Service (NHS), while boys are not. A bivalent vaccine Cervarix (which used to be used by the NHS) and a nonavalent vaccine Gardasil 9 are available privately in the UK while the NHS has used a quadrivalent vaccine Gardasil since September 2012. This quadrivalent vaccine protects against the 2 high risk HPV types (16 and 18), which are responsible for 70% of cervical cancers in addition to the aforementioned head and neck cancers and some anogenital cancers, and 2 low risk HPV types (6 and 11) responsible for 90% of genital warts. The rationale for vaccinating only girls and not boys as well is that vaccinating girls should provide herd immunity to boys. However, this is not effective for those men who have sex with the estimated 15% of girls who don’t take up the vaccine or for those who have sex with older women or women from overseas who have not been vaccinated. Most vulnerable to the negative health implications of the herd immunity rationale are men who have sex with men. As a result, there has been extensive lobbying in the UK for the HPV vaccination programme to be extended to boys. In July 2017, the UK’s Joint Committee on Vaccination and Immunisation (JCVI) published their interim statement regarding extending HPV vaccination to adolescent boys in which they state that “Clearly there is benefit in vaccinating boys and the data considered by the Committee shows that the HPV vaccine is both safe to use in boys and generates comparable immunogenicity to that seen in girls” (p20) [6]. Despite the benefit and safety of the vaccination, they conclude that “extending the HPV programme to adolescent boys would not be a cost-effective use of health service resources in the UK setting” (p21).

When considering whether to extend the vaccination to boys, there are issues to consider beyond the cost benefit (see Newman & Lacombe-Duncan [7] for some discussion). One of those issues is parental attitudes towards the vaccination. A recent qualitative study in Sweden identified that many parents interviewed were in favour of gender-neutral vaccination, however some interviews also revealed inadequate knowledge about HPV and the vaccine in particular in relation to the impact on males [8]. Similarly, a survey of parents of boys in Uganda found that 78% of parents would be willing for their sons to be vaccinated if the vaccine were available to them. Once again, knowledge was important, with parents who knew that HPV is usually sexually transmitted, that males can acquire HPV, and that HPV vaccines effectively protects against HPV more likely to say they would allow their son to be vaccinated [9].

Since the introduction of the vaccination programme for girls in 2008, there has been only one study to date exploring parental attitudes about HPV as it affects men and the male vaccination in the UK. Mortensen et al conducted a pan-European survey of parents to explore their attitudes to the vaccine and found that 75% of UK parents were in favour of their sons being vaccinated [10]. However, respondents were provided with some “brief oral information on HPV-related disease in males” (p 629) prior to answering the questions.

Additionally, although the vaccination is not yet available to boys through the NHS in the UK, it is available privately. In order for parents and their sons to avail themselves of this option, they need to be aware of the health consequences of HPV for men and to understand who might be at increased risk. To the best of our knowledge, there has been no research in the UK exploring what parents know about the health sequelae of HPV for males beyond genital warts. It is therefore unknown what parents of boys know about HPV as it relates to their sons and a lack of relevant knowledge may prevent them making informed choices about their son’s health either in the event of the vaccination programme being extended to males or indeed in the event that it is not.

The present study aimed to explore what parents of teenage boys in the UK know about HPV including how it relates to male health and whether the male HPV vaccination is something parents would want for their sons. This is the first study to explore these issues in the UK without providing parents with prior information about HPV and it is to be hoped that the findings will inform decision making about extending the HPV vaccination to boys as well as informing health literacy campaigns about HPV health sequelae in males.

## Materials and methods

### Participants and procedures

Participants consisted of 186 parents of male pupils from secondary schools in Staffordshire and Stoke-on-Trent, UK. Schools in the area were contacted via phone or email and invited to participate. Of the six schools who agreed to take part, four catered for pupils aged 11–18 years and two for pupils aged 11–16 years. Only one school had a religious affiliation (Roman Catholic). Pupil premium numbers, which can serve as a marker of socio-economic status, were above the national average for three schools and below average for the remaining three.

Schools sent parents a link to an online questionnaire via email or text, depending on the schools’ preferred contact method, with some schools also posting the link on their website. Participants were informed that the purpose of the study was to find out what parents of boys know and feel about HPV and the HPV vaccination. Participants provided informed consent and then completed the questionnaire, which usually took approximately 10 to 25 minutes to complete. To compensate them for their time, participants were offered the opportunity to be entered into a £200 prize draw. The study received approval from the School of Psychology Ethics Committee at Keele University. Data collection took place between September 2016 and January 2017.

### Measures

A self-report questionnaire was used to assess knowledge of HPV and HPV vaccination and attitudes and beliefs about HPV and HPV vaccination (the survey is publicly available here: https://osf.io/j9kgc, http://doi.org/10.17605/OSF.IO/J9KGC), it is also in S1 File). The questionnaire consisted of 4 sections: part 1 asked for basic socio-demographic information; part 2 investigated knowledge about HPV; part 3, which was only answered by those who had already heard of HPV vaccination, examined knowledge about HPV vaccination, engagement with HPV vaccination for daughters and potential engagement with HPV vaccination for sons; and part 4, which was completed by all participants, explored attitudes and beliefs about HPV and HPV vaccination in relation to sons. Questions were adapted from measures used in previous studies as detailed in the next paragraph with minor changes made to reflect differences in national guidelines or differences in methodology (e.g. online survey rather than interviews).

In addition to basic socio-demographic information, part 1 also asked participants to list the gender and age of all children in the household. Part 2 began with a filter question asking participants if they had heard of HPV. Those that responded ‘yes’ or ‘don’t know’ went on to answer subsequent questions in the section. The first four of these comprised four open text-box knowledge questions asking what the letters HPV stand for, what HPV is, how HPV is contracted and what the relationship is between HPV and cervical cancer [11]. This was followed by a general HPV knowledge scale (GK23) [12, 13], consisting of 23 items to which participants responded ‘true’, ‘false’ or ‘don’t know’.

Part 3 also began with a filter question, this time asking participants if they had heard of HPV vaccination before the survey, with those that responded ‘yes’ or ‘don’t know’ going on to answer subsequent questions in the section. The first four of these asked whether any daughters of the participants had received or would be allowed to receive the HPV vaccination [10] and were followed by a 9-item HPV vaccination knowledge scale [12,13]. Two items about recommendations by Health Canada from the original knowledge scale were not included. A filter question was then used asking how willing they would be for their own son to receive the vaccine if it were available to boys. Those that said they were ‘Definitely willing’ or ‘Probably willing’ went on to indicate the importance of 13 different reasons for their willingness on a 5-point Likert scale, whilst those who reported being ‘Definitely not willing’, ‘Probably not willing’ or ‘Not sure’ rated the importance of 14 different reasons for being unwilling or uncertain [10]. Both groups were also offered the opportunity to provide additional reasons.

Before completing part 4, participants were given the following information about HPV and HPV vaccination:

*The Human Papillomavirus (HPV) is the most common sexually transmitted infection. HPV can cause genital warts. HPV can also cause cancers of the cervix, penis, anus, vagina, vulva and oral cancers*.

*An HPV vaccine, Gardasil^®^, is currently offered to girls aged 12–13 years as part of the NHS childhood vaccination programme. The HPV vaccine is delivered largely through secondary schools, and currently consists of two injections into the upper arm (girls who began vaccination before September 2014 receive three injections)*.

*The HPV vaccination is not currently offered to boys in the UK as part of the NHS childhood vaccination programme*.

They were then asked if they believe that boys should receive the HPV vaccine (yes/no/don’t know). This was followed by 39 items from the HPV Attitudes and Beliefs Scale (HABS) [14] which mapped onto seven scales: benefits (e.g., I feel that the HPV vaccine would protect my son’s sexual health), harms (e.g., I feel that giving my son the HPV vaccine would be like performing an experiment on him), risk (e.g., I feel that without the HPV vaccine, my son would be at risk of getting genital warts later in life), threat (e.g., I feel that it would be serious if my son contracted an HPV-related cancer later in life), influence (e.g., I feel that most of my friends would think vaccinating my son against HPV is a good idea), communication (e.g., I feel that sex is not a subject I talk about with my son), and general vaccination attitudes (e.g., I feel that doctors give out too many vaccines). The tense of some questions was changed to reflect the fact that the vaccination is not currently offered to boys in the UK. Three items on affordability and four items on accessibility were not included since the vaccination is provided free of charge to girls in a school-based programme in the UK and at the time the survey was developed it was not widely available on a private basis. For each item, participants indicated their agreement on a 7-point Likert scale (1 = Strongly disagree, 4 = Neutral, 7 = Strongly agree). Participants were given a score of between 1 and 7 for each item, with negatively worded items recoded so that, for all items, high scores indicate a positive attitude or belief.

Finally, participants were asked if they would like to add any other comments and were offered the opportunity to put their details forward for a focus group and to take part in a £200 prize draw.

## Results

### Sample characteristics

Participants consisted of 186 parents of male secondary school pupils. The parents were aged between 28 and 65 years (M = 42.985, SD = 6.307) and had at least one son aged 11–18 years. Participants most commonly reported that there were 2 children aged 19 or under in the household (52.2%) and slightly more than half (55.4%) reported daughters as well as sons living in the household. Further demographic information can be seen in Table 1. No significant differences were found between those who had daughters and those who didn’t in terms of gender of the person completing the questionnaire, whether the person was in a relationship or not, whether they had a degree level education, whether their household income was above £24,999 or whether they were religious.

**Table 1:** Demographic characteristics.

	n = 186 (%)
Questionnaire completed by	
Mother	168 (90.3)
Father	13 (7.0)
Female guardian	3 (1.6)
Male guardian	2 (1.1)
Marital Status	
Married or living with partner	151 (81.2)
Single	9 (4.8)
Divorced, separated or widowed	21 (11.3)
Missing	5 (2.7)
Education level	
Postgraduate degree (Graduate school)	31 (16.7)
First degree (College graduate)	40 (21.5)
A-levels or equivalent (College)	66 (35.5)
GCSEs/O-levels or equivalent (High school)	41 (22.0)
No formal qualifications/missing	8 (4.3)
Annual Household Income	
Under £15,000	53 (28.5)
£15,000 to £24,999	50 (26.9)
£25,000 to £34,999	25 (13.4)
£35,000 to £44,999	15 (8.1)
£45,000 or more	18 (9.7)
Rather not say/missing	25 (13.4)
Ethnicity	
White	182 (97.8)
Other	4 (2.2)
Religion	
No religion	60 (32.3)
Christian	124 (66.7)
Muslim	1 (0.5)
Other	1 (0.5)
Children aged 19 or under in household	
1	37 (19.9)
2	97 (52.2)
3	39 (21.0)
4+	13 (7)
Gender of all children in household	
Male only	83 (44.6)
Female and male	103 (55.4)

### HPV awareness and knowledge

Overall, 53.2% of participants reported that they had heard of HPV before completing the survey, 45.2% had not and 1.6% didn’t know. Those participants who answered, ‘don’t know’, were excluded from further analyses in this section. Of parents who had both sons and daughters, 63.6% had heard of HPV and, of parents who had only sons, 36.4% had heard of HPV. There was a significant difference between those who had sons and daughters and those who had sons only (χ^2^(1) = 7.036, P = 0.008), with those with daughters being more likely to have heard of HPV (OR = 2.223, CI = 1.227–4.028).

Only those participants who had heard of HPV answered the remaining questions in this section (n = 99). Responses to the open text-box items were coded qualitatively and are summarised in Table 2.

**Table 2:** Responses to open text-box items for HPV awareness and knowledge.

Item and coded responses	(N = 99)
	%
**What do the letters HPV stand for?**^1^	
Human papillomavirus	59.6
**What is HPV?**^2^	
Virus	50.5
STD/STI (not specified as viral)	10.1
Vaccine	10.1
Infection (not specified as sexually transmitted or viral)	5.1
Genital warts/Cause of warts	3.0
Cervical cancer/Type of cancer	3.0
Herpes	3.0
Cause of cervical cancer	3.0
Other	4.0
Don't know/Blank	8.1
**How does someone get HPV?**^**2**^^,^^3^	
At least one response indicating sexual transmission	78.8
Sexual intercourse	32.3
Sexual contact or genital skin-to-skin contact	26.3
Sexually transmitted	18.2
Oral sex	12.1
Anal sex	5.1
Unprotected sex	5.1
Direct/skin-to-skin contact (no mention of sexual activity)	16.2
Kissing	5.1
Bodily fluids	5.1
Mother-to-baby	3.0
Injection	3.0
Don't know/Blank	10.1
**What is the relationship, if any, between HPV and cancer?**^4^	
HPV causes or increases the risk of cancer	72.7

^1^Minor spelling errors were disregarded provided the intention was clear.

^2^Responses coded to show misconceptions and gaps in knowledge as well as correct responses.

^3^Participants were asked to name all the ways that came to mind so responses do not total 100%. Responses given by <3% of participants have not been included.

^4^Answers were coded as correct if they indicated that HPV causes or increases the risk of cancer, cervical cancer or cell changes.

Internal consistency for the GK23 was found to be high with Cronbach’s α = .89. Two items were found to have low item-total correlations: ‘*HPV can cause cervical cancer*’ (0.234) and ‘*HPV can cause HIV/AIDS’* (0.274) so these were not included in further analyses. Cronbach’s α was not affected by the removal of these items. The median score for the 21 remaining items (GK21) was 14.00 (see Table 3 for item-level responses for the GK23). Median scores were identical for those who had daughters and those who did not (Mdn = 14.00).

**Table 3:** Responses to GK23 HPV knowledge items.

Item	Correct (%)	Incorrect (%)	Don't know (%)
HPV can cause cervical cancer (T)	93.9	1.0	5.1
HPV can be passed on during sexual intercourse (T)	83.8	7.1	9.1
Having many sexual partners increases the risk of getting HPV (T)	79.8	5.1	15.2
A person could have HPV for many years without knowing it (T)	79.8	0.0	20.2
Men cannot get HPV (F)	78.8	3.0	18.2
Using condoms reduces the risk of HPV transmission (T)	77.8	6.1	16.2
HPV always has visible signs and symptoms (F)	75.8	2.0	22.2
HPV is very rare (F)	71.7	5.1	23.2
HPV can cause genital warts (T)	71.7	11.1	17.2
HPV can be transmitted through genital skin-to-skin contact (T)	68.7	10.1	21.2
A person with no symptoms cannot transmit the HPV infection (F)	67.7	12.1	20.2
HPV can cause HIV/AIDS (F)	66.7	3.0	30.3
Having sex at an early age increases the risk of getting HPV (T)	62.6	15.2	22.2
HPV can be transmitted through anal sex (T)	58.6	6.1	35.4
There are many types of HPV (T)	57.6	4.0	38.4
HPV can be transmitted through oral sex (T)	56.6	9.1	34.3
HPV is a bacterial infection (F)	51.5	18.2	30.3
HPV can cause oral cancer (T)	49.5	8.1	42.4
HPV can be cured with antibiotics (F)	48.5	10.1	41.4
HPV can cause anal cancer (T)	45.5	10.1	44.4
HPV infections always lead to health problems (F)	39.4	23.2	37.4
HPV can cause cancer of the penis (T)	35.4	10.1	54.5
Most sexually active people will get HPV at some point in their lives (T)	32.3	33.3	34.3

### Vaccination awareness and knowledge

Overall, 54.8% of participants reported that they had heard of HPV vaccination before completing the survey (n = 102), 44.1% had not and 1.1% didn’t know. Those participants who responded, ‘don’t know’, were excluded from further analyses in this section. Of parents who had both sons and daughters, 61.8% had heard of HPV vaccination and, of parents who had only sons, 46.3% had heard of HPV vaccination. There was a significant difference between those who had daughters and those who had sons only (χ^2^(1) = 4.367, P = 0.037), with those with daughters being more likely to have heard of HPV vaccination (OR = 1.870, CI = 1.037–3.374).

Only those participants who had previously heard of HPV vaccination answered the remaining questions in this section. Of the 39 participants who had daughters aged 0–11 years, 76.9% intended for them to have the HPV vaccination, 10.3% did not intend to and 12.8% didn’t know. For the 29 participants with daughters aged 12–17 years, 75.9% said they had already received the vaccination, 10.3% intended to have them vaccinated, 10.3% had not and did not intend to, and 3.4% didn’t know. Of the 21 participants with daughters aged 18 years or over, 71.4% said they had received the vaccination, 23.8% had not and 4.8% didn’t know.

Item-level responses for the VK9 can be seen in Table 4.

**Table 4:** Responses to VK9 HPV vaccination knowledge items.

Item	Correct (%)	Incorrect (%)	Don't know (%)
Girls who have had the HPV vaccine do not need a smear test (cervical screening) when they are older (F)	94.1	0.0	5.9
The HPV vaccine offers protection against all sexually transmitted infections (F)	83.3	1.0	15.7
Someone who has had the HPV vaccine cannot develop cervical cancer (F)	75.5	2.0	22.5
You can cure HPV by getting the HPV vaccine (F)	65.7	3.9	30.4
The HPV vaccines offer protection against most cervical cancers (T)	53.9	15.7	30.4
The HPV vaccine protects you from every type of HPV (F)	51.0	2.9	46.1
The HPV vaccine requires at least 2 doses (T)	50.0	13.7	36.3
The HPV vaccines are most effective if given to people who've never had sex (T)	44.1	22.5	33.3
One of the HPV vaccines offers protection against genital warts (T)	27.5	18.6	53.9

Internal consistency for the VK9 was moderate (Cronbach’s α = .68). Three items were found to have low item-total correlations: ‘*The HPV vaccine requires at least 2 doses’* (0.237) and ‘*The HPV vaccines offer protection against most cervical cancers*’ (0.037) and ‘*One of the HPV vaccines offers protection against genital warts*’ (0.200) so these were not included in further analyses. This resulted in improved internal consistency (Cronbach’s α = 0.76). The median score for the remaining 6 items (VK6) was 5.0. The median score for participants with daughters (Mdn = 5.00) was slightly higher than for those with sons only (Mdn = 4.00) but a Mann-Whitney U test showed that this difference was not significant, U = 1085.00, z = -1.013, p = 0.311.

Out of the 102 participants who had previously heard of HPV vaccination, 49.0% said they would be ‘definitely willing’ for their son to receive the HPV vaccination if it was available for boys, 30.4% were ‘probably willing’, 4.9% were ‘definitely not willing’, 1.0% were ‘probably not willing’ and 14.7% were ‘not sure’. The importance ratings of various reasons for being willing to vaccinate or not willing to vaccinate are shown in Tables 5 and 6 respectively.

**Table 5:** Importance of reasons for being willing to vaccinate sons (N = 81).

Reason	Very important	Somewhat important	Neither important or unimportant	Somewhat unimportant	Very unimportant	No response
Both sexes are equally responsible for preventing sexually transmitted infections	96.3	2.5	0.0	0.0	1.2	0.0
Because my son is also at risk of HPV infection (just as girls are)	92.6	6.2	0.0	1.2	0.0	0.0
I welcome any protection of my children against cancer	90.1	8.6	0.0	0.0	1.2	0.0
To protect my son's future partners from cancer and/or genital warts	91.4	4.9	2.5	0.0	1.2	0.0
I might regret not vaccinating my son, if he later gets an HPV related disease	77.8	16.0	4.9	0.0	1.2	0.0
To protect my son against genital warts	80.2	12.3	4.9	0.0	2.5	0.0
Both sexes should have equal rights to vaccination	82.7	8.6	4.9	1.2	1.2	1.2
I welcome all vaccines for children	64.2	25.9	6.2	2.5	0.0	1.2
To protect my son against sexually transmitted infections/diseases (other than genital warts)	77.8	8.6	11.1	0.0	2.5	0.0
If HPV vaccination is recommended by the Department of Health as part of a national immunisation programme, I would vaccinate without questioning	55.6	27.2	13.6	2.5	1.2	0.0
Because/if HPV vaccination is recommended by a health care professional (e.g. GP or nurse)	48.1	29.6	19.8	1.2	1.2	0.0
Because of personal experiences with dysplasia or cancer (myself or close relations)	39.5	16.0	29.6	3.7	9.9	1.2
Because of personal experiences with genital warts (myself or close relations)	9.9	1.2	45.7	3.7	39.5	0.0

**Table 6:** Importance of reasons for being unwilling to vaccinate sons or unsure (N = 21).

Reason	Very important	Somewhat important	Neither important or unimportant	Somewhat unimportant	Very unimportant	No response
I fear side effects (incl. that the vaccine is new)	42.9	52.4	4.8	0.0	0.0	0.0
I don’t know enough about HPV vaccination	71.4	19.0	9.5	0.0	0.0	0.0
I don’t know enough about HPV related diseases (in males)	57.1	28.6	14.3	0.0	0.0	0.0
I might regret vaccinating my son, if he later experiences side effects	23.8	38.1	19.0	9.5	9.5	0.0
Lack of recommendation from healthcare professionals	14.3	47.6	23.8	4.8	9.5	0.0
I am against (too many) vaccines	28.6	23.8	23.8	9.5	14.3	0.0
It is unlikely that my son will be HPV infected	19.0	19.0	52.4	4.8	4.8	0.0
I prefer that my son makes his own decision later	9.5	28.6	23.8	14.3	23.8	0.0
It is sufficient that females are vaccinated	14.3	14.3	38.1	14.3	19.0	0.0
Pre-marital sex and HPV vaccination goes against my cultural/ religious beliefs	4.8	9.5	33.3	14.3	38.1	0.0
My son is too young–it is not yet relevant	4.8	9.5	47.6	9.5	28.6	0.0
My son is afraid of needles–does not want to see the doctor	0.0	14.3	28.6	14.3	42.9	0.0
It is too late–my son already had his first sexual experience	0.0	4.8	33.3	9.5	52.4	0.0
The (out-of-pocket) cost is too much	0.0	0.0	61.9	4.8	28.6	4.8

For those participants who were willing to vaccinate their sons against HPV (N = 81), the reasons rated as either very or somewhat important by the highest proportion of participants were, *‘Both sexes are equally responsible for preventing sexually transmitted infections’*, *‘Because my son is also at risk of HPV infection (just as girls are)*’ and *‘I welcome any protection of my children against cancer’* (all 98.8%). The reasons rated as very or somewhat unimportant by the highest proportion of participants were, ‘*Because of personal experiences with genital warts (myself or close relations)*’ (43.2%) and ‘*Because of personal experiences with dysplasia or cancer (myself or close relations)*’ (13.6%).

For those participants who were unwilling to or not sure about vaccinating their sons against HPV (N = 21), the reasons rated as either very or somewhat important by the highest proportion of participants were, *‘I fear side effects (incl*. *that the vaccine is new)*’ (95.2%), ‘*I don’t know enough about HPV vaccination’* (90.5%) and ‘*I don’t know enough about HPV related diseases (in males)’* (85.7%). The reasons rated as very or somewhat unimportant by the highest proportion of participants were, ‘*It is too late–my son already had his first sexual experience’* (61.9%) and, *‘My son is afraid of needles–does not want to see the doctor’* (57.1%). In addition, all participants rated, ‘*The (out-of-pocket) cost is too much’* as unimportant or neither important or unimportant.

Two reasons related to perceived regret: ‘*I might regret not vaccinating my son*, *if he later gets an HPV related disease’* for participants who were willing to vaccinate their sons, *and ‘I might regret vaccinating my son*, *if he later experiences side effects’* for those who were unwilling or unsure. Of parents who were willing, 93.8% rated perceived regret as either somewhat or very important. Of parents who were unwilling or unsure, 61.9% rated perceived regret as either somewhat or very important. This difference was significant (χ^2^(1) = 15.281, P = 0.037), with those who were willing being more likely to rate perceived regret as being important (OR = 9.354, CI = 2.646–33.067).

For HPV knowledge (GK21), scores were higher for parents who were willing to vaccinate their sons (Mdn = 15.0) than for parents who were unwilling or unsure about vaccinating their sons (Mdn = 9.0). A Mann-Whitney U test showed that this difference was significant, U = 431.50, z = -2.595, p = 0.009. Parents who were willing to vaccinate their sons also scored higher (Mdn = 5.0) on HPV vaccination knowledge (VK6) than parents who were unwilling or unsure about vaccinating (Mdn = 4.0). However, a Mann-Whitney U test showed that this difference was not significant, U = 725.0, z = -1.064, p = 0.287.

### HPV vaccination for boys

After reading some brief information about HPV and HPV vaccination, 85.5% (N = 159) of participants believed that boys should receive the vaccine, 3.8% (N = 7) believed that they should not and 10.8% (N = 20) said they didn’t know. These responses were compared to those given to the previous question regarding willingness of participants to vaccinate their own sons. Of the 81 participants who previously said they would be willing to vaccinate their own son against HPV, 78 also said that boys should receive the vaccine and 3 didn’t know if boys should receive the vaccine. Of the 6 participants who were previously unwilling to vaccinate their own, 4 also believed that boys shouldn’t be offered the vaccine whilst the remaining 2 thought that they should. For those that were previously unsure about vaccinating their own sons, 8 went on to say that boys should be offered the vaccine, 1 said they should not and 6 remained unsure.

For further analyses, those participants that said boys should receive the HPV vaccine are referred to as approvers and those that said they shouldn’t or didn’t know are referred to as disapprovers/doubters. For the GK21, scores were higher for approvers (Mdn = 14.0) than disapprovers/doubters (Mdn = 11.0) but a Mann-Whitney U test showed that this difference was not significant, U = 461.00, z = -1.018, p = 0.309. For the VK6, approvers scored higher (Mdn = 5.00) than disapprovers/doubters (Mdn = 4.50) but a Mann-Whitney U test showed that this difference was not significant, U = 586.00, z = -0.299, p = 0.765.

For the HABS scales, internal consistency was high for all subscales (Cronbach’s α > 0.8). Summary information and median scores can be seen in Table 7. Univariate logistic regression analyses, as seen in Table 8, showed that scores for 5 out of the 7 HABS subscales were significant predictors of approval of vaccination for boys: benefits, influence, harms, risk and general vaccination opinions. HPV knowledge and HPV vaccination knowledge were not found to be significant predictors. One item within the harms subscale was used a measure of perceived safety of the vaccine (‘I feel that the HPV vaccine is unsafe’) and this was also found to be a significant predictor of approval.

**Table 7:** Median scores for HABS subscales.

Subscale	Max Score	Cronbach's α	All participants	Approvers	Disapprovers /Doubters	p value^1^
Benefits	70	0.95	53	55	40	p < .001
Threat	21	0.91	19	19	18	p = .016
Influence	56	0.95	45	46	33	p < .001
Harms	42	0.93	27	28	23	p < .001
Risk	21	0.96	13	15	12	p = .001
Communication	35	0.91	30	30	30	p = .994
General vaccination opinions	28	0.82	24	24	19	p = .001
Total score	273		209	216	173	P < .001

^1^Mann-Whitney U Test

**Table 8:** Univariate logistic regression predicting likelihood of approval of vaccination of boys.

Variable	SE	Wald	*df*	*p*	Odds Ratio	95% CI
Benefits *(Benefits)*	0.018	19.026	1	<0.001	1.083	1.045–1.123
Threat *(Severity)*	0.048	3.043	1	0.081	1.087	0.990–1.194
Influence *(Cues to action)*	0.023	18.873	1	<0.001	1.104	1.056–1.154
Harms *(Barriers)*	0.039	19.949	1	<0.001	1.19	1.103–1.285
Risk *(Susceptibility)*	0.047	8.18	1	0.004	1.143	1.043–1.252
Communication	0.032	0.358	1	0.55	1.02	0.957–1.086
General Vaccination Opinions	0.046	12.012	1	0.001	1.171	1.071–1.281
Perceived safety item	0.215	17.039	1	<0.001	2.432	1.595–3.709
GK21 Score	0.163	0.003	1	0.955	1.009	0.734–1.388
VK6 Score	0.052	0.387	1	0.534	1.033	0.933–1.144

## Discussion

This is the first study to explore what parents of teenage boys in the UK know about HPV and its impact on male health since the introduction of the HPV vaccination programme and it is also the first to explore their attitudes towards their sons being offered the HPV vaccination without first being provided with information about HPV. The results are briefly summarized here. Only half of the parents in our survey had heard of HPV prior to the survey and the knowledge figures were similar for the HPV vaccination. For those who had heard of the vaccination, most were either definitely or probably willing for their son(s) to receive it. Once all participants had been provided with some brief information about HPV, the majority thought their sons should be offered the vaccine.

We explore these findings in more depth below.

### HPV awareness and knowledge

Only half of the parents in our survey had heard of HPV prior to the survey and this number was considerably lower for those parents who did not have daughters. Unsurprisingly given that the HPV vaccination has been available for girls since 2008, most respondents who had heard of HPV correctly identified that it causes cervical cancer, although the number identifying this link was lower in the open text answers than in the subsequent GK23 knowledge questions. However, their knowledge was much poorer for cancers that affect men, with fewer than half of respondents correctly identifying that HPV causes throat cancer or anal cancer and only a third identifying that it causes cancer of the penis. Very few respondents named oral or anal sex as ways of contracting HPV in the open text answers.

As Rasidic et al identify in their systematic review of factors associated with parents’ attitudes to the HPV vaccination of their adolescent sons, knowledge is an important factor in vaccine acceptability [15]. Our findings also reflect the importance of knowledge, with HPV knowledge scores higher for parents who were willing to vaccinate their sons than for parents who were unwilling or unsure about vaccinating their sons. Beyond acceptability, in the event that the vaccination is not made available to boys for free in the UK as it is for girls, it is unlikely that in the absence of knowledge of the implications of HPV infection for boys’ parents would proactively seek out the vaccination for their sons. While the JCVI did recommend that the HPV vaccine be made available to men who have sex with men who attend a GUM or HIV clinic [16], this is not on demand, but rather only opportunistically for those MSM attending the clinics for other health care. Since the vaccine is most effective pre-exposure and when administered pre-puberty, when the body’s immune response is strongest [17], this is too little too late.

### Vaccination awareness and knowledge

Only around half of respondents had heard of the HPV vaccination prior to this survey and again this was lower for those parents who did not have daughters. Knowledge about the vaccination was slightly better than for HPV although this was based on fewer items. However, fewer than half of respondents knew that the vaccination was more effective if given to people who have never had sex and this lack of knowledge is a worrying gap if parents are to make informed choices about the vaccination before their sons become sexually active. Despite this, for those who had heard of the vaccination, most were either definitely or probably willing for their son(s) to receive it and this is also consistent with previous research that shows that parents are generally supportive of HPV vaccination for boys (for example [18, 15]). The respondents cited health equality issues as important to their response, such as ‘Both sexes are equally responsible for preventing sexually transmitted infections’ and ‘Because my son is also at risk of HPV infection (just as girls are)’. This health equality issue has been acknowledged by the JCVI who, in their recent statement, wrote “the Committee recognises arguments made by stakeholders on the issue of equality of access and that there are additional clinical benefits that could be achieved in males with a gender neutral programme. The Committee therefore wishes to refer the issue of equality of access to the Department of Health for consideration” (p21) [6]. Our findings demonstrate how important health equality concerns are to parents and their perspective needs to feed into any decisions made about access to the HPV vaccination in the UK and elsewhere. The fourth most important reason identified for why parents would be willing for their son to receive the vaccine related to herd immunity “to protect my son's future partners from cancer and/or genital warts”. This is consistent with the findings of a review by Quadri-Sheriff et al [19] who explored whether the concept of herd immunity (vaccinating your child in order to protect others) was a motivator for parents to get their children vaccinated with childhood vaccinations such as measles/mumps/rubella (MMR), human papillomavirus (HPV) or varicella for example. While the main motivation for vaccination was to benefit their own child, in 17 qualitative studies, benefit to others was identified as a motivation to vaccination, while in 3 quantitative studies 37% of parents ranked benefit to others as their second most important factor in decision-making.

For the minority of parents in our study who were unwilling or unsure about vaccinating their sons, knowledge or lack thereof played a role in their response, with ‘I don’t know enough about HPV vaccination’ and ‘I don’t know enough about HPV related diseases (in males)’ being the most important reasons after ‘I fear side effects (incl. that the vaccine is new)’. Ten studies in the review by Rasidic et al [15] reported a consistent relationship between lack of awareness and vaccine non-acceptability. Should the JCVI decide against extending the programme to boys, this is likely to become even more important since parents will need to proactively seek out the vaccination and since the vaccination is not cheap (~£150 per dose) they would need to be even more certain that the vaccination would have a protective effect for their son’s health to be motivated to obtain it.

For both those parents willing and those unwilling or unsure about vaccinating their sons, anticipated regret was important although regret for different things (the consequences of not-vaccinating and the consequences of vaccinating respectively) but this was most important for those who were willing to vaccinate their sons.

### HPV vaccination for sons (all participants)

Once all participants had been provided with some brief information about HPV, the majority thought their sons should be offered the vaccine. We investigated predictors of acceptability using the HABS [14]. The development of the HABS was informed by several social cognition models including the Health Belief Model, the Theory of Planned behavior and the Integrated Behavioural Model [14]. Five of the seven HABS subscales we used were important for differentiating between vaccine approvers and vaccine doubters/disapprovers: benefits, influence, harms, risk and general vaccination opinions. In the Health Belief Model the first four map onto the constructs of perceived benefits, cues to action, perceived barriers and perceived risk or susceptibility respectively and Rasidic et al observe that all of these are related to vaccine acceptability of the male vaccination amongst parents [15]. As Krawcyzk et al observe, these theoretically motivated constructs are useful for targeting interventions to increase vaccination uptake or indeed for specific populations such as men who have sex with men [20]. Thus since parents who approved of the vaccination for boys felt the vaccination would be beneficial to their son, thought that friends and clinicians would favour vaccination, believed the vaccine to be safe and believed that without the vaccine their son would be at risk of HPV and related diseases, all of these elements could feature in future interventions. These findings from our parental survey chime with the findings and recommendations of a systematic review and meta-analysis exploring vaccine acceptability among men in which positive HPV vaccine attitudes, health care provider recommendation, perceived HPV risk and HPV awareness and knowledge all impact on acceptability. The authors suggest that “health promotion messaging that fosters positive attitudes about HPV vaccination benefits for men, accurate HPV risk perceptions, and that enhances awareness and knowledge regarding HPV may increase the acceptability of HPV vaccination for men” [21]. Although scores for knowledge about HPV and the HPV vaccination were not significant predictors in our survey, this is likely to be because information was provided for all parents between these scales being measured and the final question about whether boys should be offered the vaccine, thus eliminating differences between levels of knowledge.

One important difference between this study and some of the previous research exploring vaccine acceptability amongst parents of boys is that some studies have been conducted in countries where the vaccine is already widely available for boys. In contrast, since this is not the case in the UK, our study is exploring how parents would feel if it were made available. Zimet and Rosenthal conducted a review of international research exploring parental attitudes towards HPV vaccination of sons before it was widely available for males and in common with our findings, all studies reporting that a majority of parents (more than 65% in all but two studies) endorsed male vaccination [18]. In October 2009, the US Food and Drug Administration (FDA) approved the quadrivalent HPV vaccine for boys aged 9–26. Holman et al conducted a systematic review of research conducted in or after 2009 exploring barriers to adolescent HPV vaccination in the US [22]. They included parents in their review and identified that lack of knowledge about the vaccine was a barrier to uptake while the age of the child sometimes contributed to either delaying or refusing the vaccine. Cost was sometimes identified as a barrier however this would not be relevant in the UK if the vaccine were administered to boys via the same school-based system as girls in the UK. A doctor’s recommendation or discussion about HPV was associated with increased acceptability and vaccine initiation.

Our research is also comparable to some of the research conducted before the vaccination was made available to girls in 2008. Marlow et al asked mothers of teenage girls about vaccine acceptability and the figures were very similar to those reported here, with 75% of mothers saying that they would probably or definitely accept the HPV vaccine for their daughter [23]. Perez et al also identify that there are different stages of vaccine decision making, identifying that for much of the previous research “the presumption is then that parents are already aware, engaged, and have made a decision about HPV vaccination, when in fact many parents report that they are unaware of the HPV vaccine generally and that the HPV vaccine is available for their son” (p4714) [24]. In the UK, the vaccination has not been made widely available for males and as our sample demonstrate, many parents are unaware of HPV or the vaccination, placing them in a pre-contemplation stage of decision making which they can only move out of once they have been provided with relevant information [24].

There are some limitations to this study: it is relatively small-scale study and the sample might not be fully representative of the wider population, in addition the vast majority of respondents were mothers rather than fathers. The questionnaire was rather long and this may well have put some respondents off completing it. Despite this, the findings are in line with previous research and since harder to reach populations generally have poorer health related knowledge, they are likely to be less well informed than the current sample with the attendant issues outlined above being even more relevant.

### Conclusions

Only half of the parents in our study reported having heard of HPV prior to completing the survey. For those who had heard of HPV, their knowledge about the health sequelae of HPV for men was poor relative to their knowledge of its impact on female health. There is a pressing need for public education about the potential impact of HPV on male health in order to facilitate uptake of the vaccine in the event of the vaccination programme being extended to men or to facilitate informed decision making about seeking the vaccine in the event that it isn’t. Once parents are provided with knowledge, our study suggests that the majority will want the vaccination to be offered to their sons, primarily for reasons of health equality. It is vital that informed parental views are taken into account.

## Supporting information

S1 File Survey: Vaccinating boys against HPV: The parent perspective. (DOCX)

## References

[1] World Health Organization. Human papillomavirus vaccines: WHO position paper, May 2017. Weekly Epidemiological Record, 2017;92: 241–268. 28530369

[2] Walboomers JM, Jacobs MV, Manos MM, Bosch FX, Kummer JA, Shah KV, et al Human papillomavirus is a necessary cause of invasive cervical cancer worldwide. The Journal of pathology. 1999 9 1;189(1):12–9. doi: 10.1002/(SICI)1096-9896(199909)189:1<12::AID-PATH431>3.0.CO;2-F 10451482 10.1002/(SICI)1096-9896(199909)189:1<12::AID-PATH431>3.0.CO;2-F

[3] Parkin DM, Bray F. The burden of HPV-related cancers. Vaccine. 2006 8 21;24:S11–25. 10.1016/j.vaccine.2006.05.111 16949997

[4] Hartwig S, Syrjänen S, Dominiak-Felden G, Brotons M, Castellsagué X. Estimation of the epidemiological burden of human papillomavirus-related cancers and non-malignant diseases in men in Europe: a review. BMC cancer. 2012 12;12(1):30. 22260541 10.1186/1471-2407-12-30 PMC3293758

[5] Mehanna H, Jones TM, Gregoire V, Ang KK. Oropharyngeal carcinoma related to human papillomavirus. BMJ. 2010; 340: c1439– doi: 10.1136/bmj.c1439 20339160 10.1136/bmj.c1439

[6] JCVI. JCVI Interim Statement on Extending HPV Vaccination to Adolescent Boys. July 2017. Available from https://www.gov.uk/government/uploads/system/uploads/attachment_data/file/630125/Extending_HPV_Vaccination.pdf Cited 4th September 2017.

[7] Newman PA, Lacombe-Duncan A. Human papillomavirus vaccination for men: advancing policy and practice. Future Virology. 2014 12;9(12):1033–47.

[8] Gottvall M, Stenhammar C, Grandahl M. Parents' views of including young boys in the Swedish national school-based HPV vaccination programme: a qualitative study. BMJ open. 2017 2 1;7(2):e014255 doi: 10.1136/bmjopen-2016-014255 28246143 10.1136/bmjopen-2016-014255 PMC5337740

[9] Muhwezi WW, Banura C, Turiho AK, Mirembe F. Parents' knowledge, risk perception and willingness to allow young males to receive human papillomavirus (HPV) vaccines in Uganda. PloS one. 2014 9 9;9(9):e106686 doi: 10.1371/journal.pone.0106686 25203053 10.1371/journal.pone.0106686 PMC4159277

[10] Mortensen GL, Adam M, Idtaleb L. Parental attitudes towards male human papillomavirus vaccination: a pan-European cross-sectional survey. BMC Public Health. 2015 12;15(1):624. 26152138 10.1186/s12889-015-1863-6 PMC4495645

[11] Sherman SM, Nailer E, Minshall C, Coombes R, Cooper J, Redman CW. Awareness and knowledge of HPV and cervical cancer in female students: A survey (with a cautionary note). Journal of Obstetrics and Gynaecology. 2016 1 2;36(1):76–80. doi: 10.3109/01443615.2015.1041886 26408400 10.3109/01443615.2015.1041886

[12] Perez S, Tatar O, Ostini R, Shapiro GK, Waller J, Zimet G, et al Extending and validating a human papillomavirus (HPV) knowledge measure in a national sample of Canadian parents of boys. Preventive medicine. 2016 10 1;91:43–9. doi: 10.1016/j.ypmed.2016.07.017 27471023 10.1016/j.ypmed.2016.07.017

[13] Waller JO, Ostini R, Marlow LA, McCaffery K, Zimet G. Validation of a measure of knowledge about human papillomavirus (HPV) using item response theory and classical test theory. Preventive medicine. 2013 1 1;56(1):35–40. doi: 10.1016/j.ypmed.2012.10.028 23142106 10.1016/j.ypmed.2012.10.028

[14] Perez S, Shapiro GK, Tatar O, Joyal-Desmarais K, Rosberger Z. Development and validation of the human papillomavirus attitudes and beliefs scale in a National Canadian sample. Sexually transmitted diseases. 2016 10 1;43(10):626–32. doi: 10.1097/OLQ.0000000000000506 27631357 10.1097/OLQ.0000000000000506

[15] Radisic G, Chapman J, Flight I, Wilson C. Factors associated with parents’ attitudes to the HPV vaccination of their adolescent sons: a systematic review. Preventive medicine. 2017 2 1;95:26–37. doi: 10.1016/j.ypmed.2016.11.019 27932052 10.1016/j.ypmed.2016.11.019

[16] JCVI. JCVI statement on HPV vaccination of men who have sex with men. November 2015. Available from https://www.gov.uk/government/uploads/system/uploads/attachment_data/file/477954/JCVI_HPV.pdf Cited 4th September 2017.

[17] Stanley M, Lowy DR, Frazer I. Prophylactic HPV vaccines: underlying mechanisms. Vaccine. 2006 8 21;24:S106–13. 10.1016/j.vaccine.2006.05.110 16949996

[18] Zimet GD, Rosenthal SL. HPV vaccine and males: issues and challenges. *Gynecol Oncol*. 2010; 117(2 Suppl):S26–31. doi: 10.1016/j.ygyno.2010.01.028 20129653 10.1016/j.ygyno.2010.01.028

[19] Quadri-Sheriff M, Hendrix KS, Downs SM, Sturm LA, Zimet GD, Finnell SM. The role of herd immunity in parents’ decision to vaccinate children: a systematic review. Pediatrics. 2012 9 1;130(3):522–30. doi: 10.1542/peds.2012-0140 22926181 10.1542/peds.2012-0140

[20] Krawczyk A, Knäuper B, Gilca V, Dubé E, Perez S, Joyal-Desmarais K, et al Parents’ decision-making about the human papillomavirus vaccine for their daughters: I. Quantitative results. Human vaccines & immunotherapeutics. 2015 2 1;11(2):322–9. 25692455 10.1080/21645515.2014.1004030 PMC4514251

[21] Newman PA, Logie CH, Doukas N, Asakura K. HPV vaccine acceptability among men: a systematic review and meta-analysis. Sex Transm Infect. 2013 11 1;89(7):568–74. doi: 10.1136/sextrans-2012-050980 23828943 10.1136/sextrans-2012-050980 PMC3812849

[22] Holman DM, Benard V, Roland KB, Watson M, Liddon N, Stokley S. Barriers to human papillomavirus vaccination among US adolescents: a systematic review of the literature. JAMA pediatrics. 2014 1 1;168(1):76–82. doi: 10.1001/jamapediatrics.2013.2752 24276343 10.1001/jamapediatrics.2013.2752 PMC4538997

[23] Marlow LA, Waller J, Wardle J. Parental attitudes to pre-pubertal HPV vaccination. Vaccine. 2007 3 1;25(11):1945–52. doi: 10.1016/j.vaccine.2007.01.059 17284337 10.1016/j.vaccine.2007.01.059

[24] Perez S, Tatar O, Gilca V, Shapiro GK, Ogilvie G, Guichon J, et al Untangling the psychosocial predictors of HPV vaccination decision-making among parents of boys. Vaccine. 2017 8 24;35(36):4713–21. doi: 10.1016/j.vaccine.2017.07.043 28757059 10.1016/j.vaccine.2017.07.043

