# Supplementary material for: Attitudes towards and knowledge about Human Papillomavirus (HPV) and the HPV vaccination in parents of teenage boys in the UK
Source: PLoS One. 2018 Apr 11;13(4):e0195801. doi: 10.1371/journal.pone.0195801 (PMC5895045; doi:10.1371/journal.pone.0195801)
Supplement: S1 File — (DOCX) [file pone.0195801.s001.docx]

**Vaccinating boys against HPV: the parent perspective**

**1. Consent form**

I confirm that I have read and understand the information page for this study. I understand that my participation is voluntary and that I don't have to complete the survey. I understand that the data collected about me will be anonymised before it is submitted for publication. I agree for any quotes to be used (they will be anonymised). I agree to allow the data collected to be used for research projects related to this project. *

Please tick the box to agree to the above statements and to take part in the study.

***2. PART 1: About you**

The following questions are important in allowing us to identify targets for future public health education. Please ensure you have answered all questions on a page as accurately as possible before moving on to the next page. Once you have click 'next page', you will not be able to return to the previous questions. Please remember that all responses are anonymous.

Who is completing this questionnaire? *

Mother

Father

Female guardian

Male guardian

Other (please specify):

What is your date of birth? *

DD/MM/YYYY

What is your marital status?

Married or living with partner

Single

Divorced, separated or widowed

What is the highest level of qualification that you have received from school, college or since leaving education?

Postgraduate degree

First degree

A-levels or equivalent

GCSEs/O-levels or equivalent

No formal qualifications

Other qualification, please specify:

What is your occupation?

What is your annual income before tax and national insurance?

Under £15,000

£15,000 to £24,999

£25,000 to £34,999

£35,000 to £44,999

£45,000 or more

Rather not say

Other (please specify):

What is your ethnic group? The categories indicated below reflect the categories used in the Census 2011. Please choose option which best describes your ethnic group or background.

• White

English/Welsh/Scottish/Northern Irish/British

Irish

Gypsy or Irish traveller

Any other white background, please specify below.

• Mixed/Multiple ethnic

White & Black Caribbean

White & Black African

White & Asian

Any other mixed background, please specify below.

• Asian/Asian British

Indian

Pakistani

Bangladeshi

Chinese

Any other Asian background, please specify below.

• Black/Africa/Caribbean/Black British

African

Caribbean

Any other black background, please specify below.

• Other Ethnic

Arab

Any other ethnic group, please specify below.

If other, please specify:

What is your religion?

No religion

Christian

Buddhist

Hindu

Jewish

Muslim

Sikh

Any other religion, please describe

What is the age and gender of all children in the household? Please start from the oldest to the youngest.

Age (in years) Gender (male/female)

Child 1

Child 2

Child 3

Child 4

Child 5

Child 6

If you have more than 6 children, please state how many

**3. PART 2: About HPV**

Please answer these questions in as much detail as you can. Please don't look the answers up as we are keen to find out what the general public really know about HPV.

Had you heard of HPV before this survey? *

Yes

No

Don't know

**4. PART 2: About HPV (continued)**

What do the letters HPV stand for?

What is HPV?

How does someone get HPV? Please name all the ways that come to mind.

What is the relationship, if any, between HPV and cancer?

**5. Part 2: About HPV (continued)**

Please answer the following questions to the best of your ability.

True False Don't know

HPV is very rare

HPV always has visible signs or symptoms

HPV can cause cervical cancer

HPV can be transmitted through genital skin-to-skin contact

There are many types of HPV

HPV can cause HIV/AIDS

HPV can be passed on during sexual intercourse

HPV can cause genital warts

Men cannot get HPV

Using condoms reduces the risk of HPV transmission

HPV can be cured with antibiotics

Having many sexual partners increases the risk of getting HPV

Most sexually active people will get HPV at some point in their lives

A person could have HPV for many years without knowing it

Having sex at an early age increases the risk of getting HPV

HPV can cause anal cancer

HPV is a bacterial infection

HPV can be transmitted through oral sex

HPV can cause cancer of the penis

HPV can be transmitted through anal sex

HPV infections always lead to health problems

HPV can cause oral cancer

A person with no symptoms cannot transmit the HPV infection

**6. PART 3: About the HPV vaccination**

Had you heard of the HPV vaccination before this survey? *

Yes

No

Don't know

**7. PART 3: About the HPV Vaccination**

If you have a daughter aged 0-11 years, will you let your daughter receive the HPV vaccination when she reaches the eligible age group of 12-13 years? *

Yes

No

Don't know

I don't have a daughter aged 0-11 years

If you have a daughter aged 12-17 years, has your daughter received the HPV vaccination? *

Yes

No

Don't know

I don't have a daughter aged 12-17 years

**8. PART 3: About the HPV Vaccination (continued)**

Do you intend to allow your daughter aged 12-17 years to have the HPV vaccination? *

Yes

No

Don't know

**9. PART 3: About the HPV Vaccination (continued)**

If you have a daughter aged 18 or older, has she received the HPV vaccination? *

Yes

No

Don't know

I don't have a daughter aged 18 or older

Please answer the following questions to the best of your ability.

True False Don't know

The HPV vaccine requires at least 2 doses

The HPV vaccine offers protection against all sexually transmitted infections

The HPV vaccines are most effective if given to people who've never had sex

Someone who has had the HPV vaccine cannot develop cervical cancer

The HPV vaccines offer protection against most cervical cancers

One of the HPV vaccines offers protection against genital warts

Girls who have had the HPV vaccine do not need a smear test (cervical screening) when they are older

The HPV vaccine protects you from every type of HPV

You can cure HPV by getting the HPV vaccine

If you have more than one son, please answer the following questions in relation to the youngest son who attends the school that asked you to complete this survey.

How willing would you be for your son to receive the HPV vaccine if it was available for boys? *

Definitely not willing

Probably not willing

Not sure

Probably willing

Definitely willing

**11. PART 3: About the HPV Vaccination (continued)**

How important is each of the items below regarding why you would be willing for your son to receive the HPV vaccine?

Very important Somewhat important Neither important nor unimportant Somewhat unimportant Very unimportant

Because my son is also at risk of HPV infection (just as girls are)

Both sexes should have equal rights to vaccination

Both sexes are equally responsible for preventing sexually transmitted infections

I welcome all vaccines for children

I welcome any protection of my children against cancer

To protect my son against genital warts

To protect my son against sexually transmitted Infections/diseases (other than genital warts)

To protect my son's future partners from cancer and/or genital warts

Because of personal experiences with dysplasia or cancer (myself or close relations)

Because of personal experiences with genital warts (myself or close relations)

Because/if HPV vaccination is recommended by a health care professional (e.g. GP or nurse)

If HPV vaccination is recommended by the Department of Health as part of a national immunisation programme, I would vaccinate without questioning

I might regret not vaccinating my son, if he later gets an HPV related disease

If you can think of other advantages to vaccinating boys that would influence your decision please specify

**12. PART 3: About the HPV Vaccination (continued)**

How important is each of the items below regarding why you would be unwilling or uncertain about your son receiving the HPV vaccine?

Very important Somewhat important Neither important nor unimportant Somewhat unimportant Very unimportant

I don’t know enough about HPV related diseases (in males)

I don’t know enough about HPV vaccination

It is sufficient that females are vaccinated

It is unlikely that my son will be HPV infected

I fear side effects (incl. that the vaccine is new)

I am against (too many) vaccines

Pre-marital sex and HPV vaccination goes against my cultural/ religious beliefs

My son is too young – it is not yet relevant

It is too late – my son already had his first sexual experience

I prefer that my son makes his own decision later

My son is afraid of needles – does not want to see the doctor

Lack of recommendation from healthcare professionals

The (out-of-pocket) cost is too much

I might regret vaccinating my son, if he later experiences side effects

If you can think of other disadvantages to vaccinating boys that would influence your decision please specify

**13. PART 4: About your son and HPV**

The Human Papillomavirus (HPV) is the most common sexually transmitted infection. HPV can cause genital warts. HPV can also cause cancers of the cervix, penis, anus, vagina, vulva and oral cancers. An HPV vaccine, Gardasil®, is currently offered to girls aged 12-13 years as part of the NHS childhood vaccination programme. The HPV vaccine is delivered largely through secondary schools, and currently consists of two injections into the upper arm (girls who began vaccination before September 2014 receive three injections). The HPV vaccination is not currently offered to boys in the UK as part of the NHS childhood vaccination programme. Do you believe boys should receive the HPV vaccine? *

Yes

No

Don't know

The following sections will present a series of statements about HPV and the HPV vaccine. We wish to know your opinion. Please note that we are not testing your knowledge. If you do not know an answer, that’s alright, simply select the answer that most reflects your opinion. If you have more than one son, please answer the following questions in relation to the youngest son who attends the school that asked you to complete this survey.

| Strongly Disagree | Disagree | Somewhat Disagree | Neutral | Somewhat Agree | Agree | Strongly Agree |
| --- | --- | --- | --- | --- | --- | --- |
| 1 | 2 | 3 | 4 | 5 | 6 | 7 |

I feel that the HPV vaccine has many benefits

I feel that the HPV vaccine would protect my son’s sexual health

I feel that the HPV vaccine works well

I feel that the HPV vaccine is effective in preventing HPV

I feel that the HPV vaccine is effective in preventing genital warts

I feel that vaccinating my son against HPV would be a good thing to do for his health

I feel that vaccinating my son against HPV would give me peace of mind about his sexual health

I feel that the HPV vaccine is effective in preventing HPV-related cancers

I feel that vaccinating my son against HPV would protect his current/future partner from getting infected with HPV

I feel that getting my son the HPV vaccine would protect his current/future partner against cancer

I feel that it would be serious if my son contracted HPV later in life

I feel that it would be serious if my son contracted genital warts later in life

I feel that it would be serious if my son contracted an HPV-related cancer later in life

I feel that other parents in my community would get their sons vaccinated against HPV

I feel that my friends would get their sons vaccinated against HPV

I feel that other boys around my son’s age would be vaccinated for HPV

I feel that it would be expected of me that I should vaccinate my son against HPV

I feel that most of my friends would think vaccinating my son against HPV is a good idea

I feel that doctors/health care providers believe vaccinating boys against HPV would be a good idea

I feel that my son's other parent would believe we should get the HPV vaccine for my son

I feel that my family would think it is a good idea to vaccinate my son against HPV

**14. PART 4: About your son and HPV**

The following sections will present a series of statements about HPV and the HPV vaccine. We wish to know your opinion. Please note that we are not testing your knowledge. If you do not know an answer, that’s alright, simply select the answer that most reflects your opinion. If you have more than one son, please answer the following questions in relation to the youngest son who attends the school that asked you to complete this survey.

| Strongly Disagree | Disagree | Somewhat Disagree | Neutral | Somewhat Agree | Agree | Strongly Agree |
| --- | --- | --- | --- | --- | --- | --- |
| 1 | 2 | 3 | 4 | 5 | 6 | 7 |

I feel that the HPV vaccine is unsafe

I feel that giving my son the HPV vaccine would be like performing an experiment on him

I feel that the HPV vaccine may lead to long-term health problems

I feel that the HPV vaccine is being pushed to make money for pharmaceutical companies

I feel that the HPV vaccine is too new

I feel that there has not been enough research done on the HPV vaccine

I feel that without the HPV vaccine, my son would be at risk of getting HPV later in life

I feel that without the HPV vaccine, my son would be at risk of getting genital warts later in life

I feel that without the HPV vaccine, my son would be at risk of getting an HPV-related cancer later in life

I feel that it is hard to talk to my son about his sexual health

I feel that I am uncomfortable discussing my son's sexual health with a doctor/health care provider

I feel that sex is not a subject I talk about with my son

I feel that I would be uncomfortable talking to my son about the HPV vaccine

I feel that I would not know how to approach the topic of the HPV vaccine with my son

I feel that vaccines are a good way to protect public health

I feel that vaccinating children is a good idea

I do not like the idea of vaccines

I feel that doctors give out too many vaccines

15. Any other comments

Would you like to add any other comments about HPV vaccination?
